# Post-Surgical Peritoneal Scarring and Key Molecular Mechanisms

**DOI:** 10.3390/biom11050692

**Published:** 2021-05-05

**Authors:** Sarah E. Herrick, Bettina Wilm

**Affiliations:** 1 Manchester Academic Health Science Centre, School of Biological Sciences, Faculty of Biology, Medicine and Health, University of Manchester, Manchester M13 9PT, UK; 2 Institute of Systems, Molecular and Integrative Biology, University of Liverpool, Liverpool L69 3BX, UK; b.wilm@liverpool.ac.uk

**Keywords:** peritoneum, mesothelium, serosal repair, post-surgical adhesions, molecular signatures, biomarkers

## Abstract

Post-surgical adhesions are internal scar tissue and a major health and economic burden. Adhesions affect and involve the peritoneal lining of the abdominal cavity, which consists of a continuous mesothelial covering of the cavity wall and majority of internal organs. Our understanding of the full pathophysiology of adhesion formation is limited by the fact that the mechanisms regulating normal serosal repair and regeneration of the mesothelial layer are still being elucidated. Emerging evidence suggests that mesothelial cells do not simply form a passive barrier but perform a wide range of important regulatory functions including maintaining a healthy peritoneal homeostasis as well as orchestrating events leading to normal repair or pathological outcomes following injury. Here, we summarise recent advances in our understanding of serosal repair and adhesion formation with an emphasis on molecular mechanisms and novel gene expression signatures associated with these processes. We discuss changes in mesothelial biomolecular marker expression during peritoneal development, which may help, in part, to explain findings in adults from lineage tracing studies using experimental adhesion models. Lastly, we highlight examples of where local tissue specialisation may determine a particular response of peritoneal cells to injury.

## 1. Introduction

Adhesions are bands of scar tissue connecting opposing organs together or to the inner abdominal cavity wall. They can take several forms, ranging from thin translucent films to thick, organised cords; however, histologically they are well vascularised, innervated with varying degrees of adipose tissue, dense collagen foci and inflammatory cells [1,2,3]. Although surgery is the main inducer of adhesions, they can also occur after inflammatory episodes such as peritonitis and endometriosis. Many patients will be clinically asymptomatic post-surgery; however, others will experience high morbidity, significant health issues, and possible hospitalisation for adhesion-related complications including bowel obstruction, female infertility, and chronic pelvic pain as well as difficulties with any repeat surgery [4]. It is proposed that adhesions form in 79–90% of patients who have open abdominal or pelvic surgery [5,6]. The Surgical and Clinical Adhesions Research (SCAR) group performed the first large retrospective cohort study of over 21,000 patients who underwent open abdominal or pelvic surgery [7]. They found that 5.7% of re-admissions were directly related to adhesions with nearly a quarter of these being in the first year after surgery [7]. Several follow-on studies have since confirmed the impact of adhesions and related complications on the number of hospital re-admissions [8,9]. Of note, although minimally invasive laparoscopy reduces the incidence of adhesion-related readmissions, overall adhesions still represent a significant burden post-surgery and further assessment suggests that the proportion of re-admissions directly related to adhesions post-surgery may have been underestimated in initial studies [10]. Furthermore, an increase in the ageing population and associated rise in metabolic disorders, such as diabetes and obesity, is proposed to be related with a higher risk of post-operative adhesion development in such patient cohorts [11]. Subsequently, adhesion associated complications impose a substantial economic burden on health systems with costs likely to increase in the future [12]. Adhesiolysis is a common surgical procedure to remove adhesions in later surgery; however, it carries a risk of bowel perforation [13], and adhesions are likely to reform at a later date [14], and so it may not be an ideal long-term therapeutic solution.

Small bowel obstruction following laparotomy constitutes between 0.9–2% of all general surgical admissions [15], and data suggests that a high percentage of these are due to adhesions [16]. A further major complication of adhesions is female infertility where bands of scar tissue distort reproductive organs so preventing normal movement of the oocyte [17]. Data has shown that women with pelvic adhesions who underwent adhesiolysis had a greater pregnancy success rate compared to women who were treated non-surgically [18]. Additional findings suggest an association between patients with peritoneal adhesions and chronic pelvic pain [14]. Indeed this may be an under-estimated problem due to difficulties in monitoring pain and the relatively rapid re-formation of adhesions after lysis and return of pain symptoms [19,20]. Furthermore, the finding that adhesions contain sensory nerves [21], and that laparoscopic manipulation of adhesions in conscious patients can elicit pain, adds further strength to a possible relationship between adhesions and chronic pain [22].

Preventative measures to reduce adhesion formation are limited but atraumatic surgical technique still represents the gold standard in combination with avoiding unnecessary handling of the viscera, meticulous haemostasis, and avoiding air drying of exposed tissues and cauterisation where necessary [23]. Preventative therapies include physical barriers that can be grouped as fluids, films and gels, and which work by keeping damaged abdominal serosa separated during the healing period. However, many of these barriers have limitations, in particular, in minimally invasive laparoscopic surgery, and uptake by surgeons is relatively low [24]. New barriers are being developed and mainly involve fabrication of natural and synthetic hydrogels (reviewed in [4]). However, preventing as many adhesions forming as possible may not necessarily relate to a proportional reduction in the risk of adhesion-related complications. For instance, it has been noted that extensive dense adhesions spanning many organs may not necessarily be linked with any complications whereas a single adhesive band could cause life-threatening bowel obstruction [25].

The combination of unmet clinical need and high health costs demonstrates the importance of pursuing a more detailed understanding of how adhesions develop in order to find improved preventative strategies to reduce their incidence. While the focus of this review is limited to the peritoneum, adhesions also occur in the other two body cavities, pleural or pericardial, and so common mechanisms may be involved. Furthermore, as the majority of adhesions occur post-operatively, the consequences of surgically related injury will be the emphasis of this review; however, there is likely overlap with other damage inducing scenarios involving chronic inflammation, infection, peritoneal dialysis, and ischaemia.

## 2. Serosal Repair and Adhesion Formation

Adhesions are generally viewed as a sequel to the normal repair process following an injury to the peritoneum. However, the reasons why adhesions occur as opposed to normal repair and serosal regeneration are still being elucidated.

### 2.1. Serosal Repair

Peritoneum is an extensive thin layer of serosa covering most internal abdominal/pelvic organs and mesentery (visceral peritoneum) and the inner body wall (parietal peritoneum), making it the largest serous membrane found in humans [26]. It consists of a surface mesothelial cell layer attached to a basal lamina overlying a vascularised submesothelial stroma consisting of mesenchymal cells including fibroblasts, endothelial cells, and immune cells. A limited amount of peritoneal fluid or plasma transudate coats the peritoneal surface [27]. This layer is essential for maintaining intra-abdominal homeostatic equilibrium and acts as a protective barrier and support for organs. Mesothelial cells show a complex organization displaying apical-basal polarity, intercellular junctions, and apical microvilli with the occasional cilia [28]. Interestingly, mesothelial cells in the adult peritoneum express both intermediate filaments of epithelial (cytokeratin) and mesenchymal (vimentin) characteristics [29]. Furthermore, cuboidal and squamous phenotypes have been observed depending on anatomical location and state of activation [30,31,32,33]. A glycocalyx produced apically consists of a carbohydrate-rich layer of proteoglycans and glycosaminoglycans which in combination with surfactant aids in lubricating serosal surfaces to reduce friction and enable smooth gliding of organs within the cavity [34,35]. Junctional complexes between cells include tight, adherens and gap junctions and desmosomes. Gap junctions act predominately as aqueous intracellular channels while adherens give structural support and tight junctions provide semipermeable properties regulating water, ions, and other solute diffusion [36]. These specialised cells perform a multitude of functions including selective fluid and cell transport, regulation of extracellular matrix (ECM) turnover through production of matrix metalloproteases (MMPs) and their inhibitors (TIMPs) as well as generation of procoagulant and fibrinolytic activity [37]. Furthermore, mesothelial cells are integral to immune induction, modulation, and inhibition and can phagocytose pathogens and present antigens to T cells [38]. Mesothelium is often overlooked and thought of as just a barrier, but much evidence now suggests that it is physiologically active responding to changes in the local environment and in fact determines the outcome of injury and/or disease related events in serosal cavities [37].

Mild injury to the peritoneum induces the release of damage-associated molecular patterns (DAMPs) from dying cells causing a rapid recruitment of innate immune cells. In particular, a strong influx of neutrophils and macrophages populate the damaged serosa within a few hours [39]. In response to a sterile injury in the liver of mice, a population of fully mature peritoneal cavity macrophages expressing the zinc finger-containing transcription factor GATA-binding protein 6 (GATA6; named after the ‘A/T**GATA**A/G’ DNA consensus motif in the regulatory regions of target genes to which all GATA family members bind) was shown to swiftly invade into areas of damage via direct recruitment across the mesothelium [40]. Such invasion was dependent on CD44 binding of macrophages to exposed hyaluronan at the injury site and ATP production providing DAMP signals from necrotic cells. Similar to other damaged epithelia, mesothelial cells at the wound edge initially lose apical-basal polarity, detach from the basal lamina and each other through degradation of junctional complexes, and start to migrate into the wound [41,42]. Indeed, an increase in mesothelial cell proliferation at the wound edge has been documented [43]. Of interest, mesothelial cells are also proposed to detach from adjacent surfaces post-injury, free-float in serosal fluid, and implant and adhere onto denuded sites where they form islands of cells that connect together to complete re-epithelialisation [44,45]. Small and large wounds are proposed to re-epithelialise in the same timeframe suggesting that at least a combination of inward migration of mesothelial cells from the wound edge and incorporation of free-floating cells is involved in the repair process. However, additional sources of new repair cells have been put forward such as mesenchymal precursors [46] and macrophages [47]. Further evidence suggests that surgical injury to the mesothelial layer induces recruitment of a circulating progenitor cell population derived from the bone marrow [48]. These progenitor cells express mesothelial markers such as the cell surface glycoprotein, mesothelin, and cytokeratins and can free-float and populate denuded serosa but only act as a temporary covering that lasts up to a month. It is proposed that these cells are the same or similar to those found by others and known as tissue repair cells [49], post-surgical cells [50], and circulating cells of hemopoietic origin [51]. It is unlikely that just one of these events is the sole mechanism by which mesothelial-like cells repopulate a denuded serosal wound, but rather several of these processes contribute to epithelial regeneration.

Strong evidence also supports the notion that mesothelial cells undergo a process of epithelial-mesenchymal transition (EMT), known as mesothelial-mesenchymal transition (MMT), following serosal damage [44,52,53]. This phenomenon has been a particular focus of the peritoneal dialysis field and thought to underlie the development of peritoneal thickening and fibrosis associated with repeated injury of the mesothelial layer [54]. Initial stages of MMT involve loss of apical-basolateral polarity combined with a reduction of epithelial cytoskeletal markers and cell-cell and cell-basal lamina detachment. Transitioned cells take on a motile phenotype and display an increase in mesenchymal cytoskeletal markers eventually becoming invasive and fully transitioned into alpha-smooth muscle actin (αSMA)- positive myofibroblasts [55]. The role of TGF-β1 in promoting MMT is well-documented [56,57,58] and several key signalling pathways associated with TGF-β, IL-1β, angiotensin II, HIF-1α, and pleiotrophin have been implicated [59,60,61,62,63]. By inducible genetic fate mapping, Chen and colleagues reported that sodium hypochlorite injured serosa is repaired by mesothelial cells positive for Wilms’ tumour protein 1 (WT1); however, they questioned the role of MMT in mediating peritoneal fibrosis [64]. Although both mesothelial cells and subserosal fibroblasts expressed αSMA in culture, only subserosal fibroblasts expressed this myofibroblast marker after chemically induced injury or overexpression of TGF-β1 in vivo. Furthermore, only subserosal fibroblasts but not mesothelial cells expressed the PDGF receptor, and inhibition of this receptor reduced peritoneal fibrosis but not re-mesothelialisation [64]. We purified rat omental mesothelial cells using an antibody against HBME1 which is expressed on mesothelial cell microvilli and found these cells to be 90% positive for WT1, and expressed transcripts of epithelial markers, *tight junction protein 1* (*Tjp1*, also known as *ZO1*), *mesothelin*, *uroplakin 3b,* and *podoplanin* but also the mesenchymal transcript, *vimentin* [65], as shown by others [29]. Interestingly, TGF-β1-treated mesothelial cells displayed a distinctive MMT signature, with downregulation of molecules involved in insulin-like growth factor (IGF) and bone morphogenic protein (BMP) signalling and upregulation of transcription factor, *Sox9* and ECM glycoproteins, *tenascin C* and *N* [65]. Genetic lineage tracing of WT1-expressing mesothelial cells after surgical injury in mice confirmed that mesothelial cells adopt a mesenchymal phenotype and migrate into the subserosa. Addition of BMP4 prevented this transition possibly highlighting a new therapeutic strategy to limit MMT and peritoneal fibrosis. A hallmark of MMT is considered the downregulation of E-cadherin and destabilisation of adherens junctions [66]; however, several groups have found low expression of this epithelial cell-cell junction protein in mesothelial cells, both in rodents and humans [65,67,68]. Variations in the state of mesothelial cell activation, possibly dependent on whether in vitro or in vivo and/or source as well as type of stimulation, may determine a heterogeneity in the MMT gene expression signature found.

### 2.2. Mechanism of Adhesion Formation

Coagulation involving platelet aggregation in combination with plasma protein deposition is part of the normal repair process. However, when this provisional fibrin-rich matrix is not cleared in a timely manner, it acts as a scaffold for tissue repair cell ingrowth and leads to increased collagen deposition and fibrosis [69]. A major contributor to adhesion development after surgical damage is the dysregulation of fibrinolysis in which levels of the activated fibrinolytic protease, plasmin, determine the balance between fibrin deposition and degradation. Plasminogen activator inhibitor-1 (PAI-1) is one of the main inhibitors of fibrinolysis whereas plasminogen activators (PAs) activate plasminogen and mediate fibrinolysis. Post-surgery, PAI-1 levels have been found to increase, and PA levels decrease, leading to delayed fibrin matrix dissolution [70]. Consequently, fibrin-rich bridges that form between closely opposed damaged serosa may persist longer than necessary, allowing an influx of repair cells. With subsequent vascularisation and ECM deposition, these fragile structures become permanent features, often within a week of surgery [71,72]. Presence of foreign body material, such as sutures or mesh implants, and/or infection will exacerbate this imbalance in fibrinolysis resulting in excessive fibrin deposition and an increased risk of adhesion development. Many studies have demonstrated that reducing tissue plasminogen activator (tPA) or increasing PAI-1 results in increased adhesion formation whereas increasing tPA and decreasing PAI-1 activity induces less adhesions to form [73,74,75,76]. Of relevance, Hellebrekers and colleagues found that in patients undergoing pelvic surgery, preoperative high PAI-1 and low tPA levels were positively correlated with extent of postoperative adhesion development [77].

The degree of inflammation is also integral to the repair outcome post-surgery. Similar events to those observed following mild serosal injury occur, with an early influx of inflammatory cells producing a plethora of growth factors and cytokines to induce subsequent granulation tissue formation and re-epithelialisation. Interestingly, cells of the adaptive immune system have been found to be essential regulators of post-surgical adhesion formation and dependent on CD28 T cell costimulatory and inhibitory programmed death-1 pathways [78,79]. Tsai and colleagues demonstrated that following a surgical injury, mesothelial cells directly mediate an influx of neutrophils and monocytes by upregulating the expression of cytokines such as CXCL1 and MCP-1 [80]. Neutrophils subsequently underwent cell senescence and formed neutrophil extracellular traps (NETosis) whereas tissue resident macrophages disappeared suggesting a change in inflammatory cell kinetics during adhesion formation [80]. Several growth factors and cytokines have been implicated in adhesion formation including VEGF, IL-6, IL-17, and IFN-γ. Again, the importance of TGF-β in post-operative adhesion development has been extensively highlighted from in vivo studies and it is well known that TGF-β1 upregulates PAI-1 production and is a major inducer of collagen deposition. Indeed, mice with only one copy of the *Tgfb1* gene develop less extensive adhesions [81] and neutralisation of TGF-β1 reduced the extent of adhesions after surgery [82,83]. Furthermore, patients with adhesions display almost double the amount of active peritoneal TGF-β1 compared with patients without adhesions [84], and this was associated with a reduced fibrinolytic activity. Torres and colleagues also found that plasma levels of preoperative TGF-β and C-reactive protein and Neutrophil-to-Lymphocyte ratio may be robust predictors of peritoneal adhesion formation in patients who had undergone previous surgery [85]. Intriguingly, the ratio of TGF-β3, another TGF-β isoform, to TGF-β1, and the spatial distribution of these two isoforms, has been found to be important in determining whether fibrous adhesions form, with high levels of TGF-β3 found to be anti-fibrotic [86]. In addition, co-localisation of the isoforms with either membrane or soluble TGF-β receptor, betaglycan, is thought to regulate adhesion development and it is proposed that different serosal tissue may predispose to forming adhesions based on their basal expression of these factors [87].

Over the last few years, there has been little advance in the development of new anti-adhesion therapies progressing into standard clinical practice. A multitude of anti-adhesion drugs have been investigated in vivo, targeting key events in adhesion pathogenesis such as coagulation/fibrinolysis, inflammation, collagen deposition, and hypoxia [4]. Unresolved issues include side effects such as bleeding, half-life of drugs in the peritoneal cavity, their localisation at the site of adhesion formation and disruption of normal wound healing at incision sites. Another approach is to develop better barrier materials that have specific properties such as: (i) biocompatible with low immunogenicity, (ii) biodegradable, (iii) easy to administer, and (iv) remaining in place over the critical stages of adhesion formation (3–5 days post-surgery). Currently available solutions, such as films and gels, are classed as physical barriers that mechanically keep serosa apart. To successfully drive therapeutic applications forward, there is a need to develop tailored barriers with properties that can regulate molecular pathways and cell behaviour, therefore modulating adhesion formation. The formulation of such applications is an exciting future prospect that requires a multi-disciplinary approach, bringing together cell biologists, pharmacologists, and material scientists. Drugs incorporated into hydrogels are appearing as a favourable option, as they act as physical barriers and can be fabricated to deliver bioactive agents in a controlled and sustained manner to affect adhesion formation [4]. Lastly, cell sheets of autologous mesothelial cells and peritoneal patches have also been assessed as anti-adhesion strategies in animal models with some promising results [88,89]. However, while a biopharmaceutical and cellular therapeutic route to regulate adhesion development is desirable, the role of peritoneal and other cells in post-surgical fibrinogenic events needs to be elucidated.

### 2.3. Cellular Contribution to Adhesions

Lineage tracing studies in mice have shown that several major peritoneal cell types contribute to adhesion formation including fibroblasts, macrophages, and mesothelial cells. Fibroblasts, specifically myofibroblasts, are known to populate adhesion tissue derived from patients [1]. Rout and colleagues found that fibroblasts isolated from adhesions and grown in culture displayed a different expression profile compared with those grown from healthy peritoneum, with genes involved in fibroblast activation and fibrogenesis were upregulated in adhesion-derived fibroblasts [90]. Moreover, targeting downstream, fibrosis-associated pathways such as Mitogen-activated protein kinase kinase (MEK) demonstrated a reduction in adhesion formation in mice [91]. Of importance, Foster et al. analysed adhesions from patients and mouse models to elucidate the heterogeneity and source of fibrogenic cells. Fibroblasts within adhesions were found to express platelet derived growth factor receptor alpha (PDGFRA), a transmembrane receptor tyrosine kinase, as well as fibroblast markers, such as αSMA, vimentin, and collagen 1 (COL1) [92]. In murine ischaemic tissue-induced adhesions, fibroblasts expressed JUN (proto-oncogene, named after viral homolog v-jun in avian sarcoma virus 17), which is a transcriptional master regulator of fibrogenesis, and a portion of these cells were also positive for the mesothelial marker mesothelin (MSLN). The authors excluded mesothelial cells as a major source of the adhesion fibroblasts using mesothelial (WT1)-specific lineage tracing in combination with the adhesion induction. Instead, they determined that tissue-resident, progenitor-type fibroblasts that proliferate polyclonally were the main contributor. Such repair cells were mainly derived from the visceral rather than parietal peritoneum. Using single cell RNA sequencing, heterogeneity between PDGFRA+ adhesion fibroblasts at early timepoints post-surgery was identified, since three transcriptionally distinct fibroblast clusters were found in mouse adhesion samples whereas four were present in human samples. Analysis of these clusters in relation to their timepoints revealed that early JUN activation promoted a profibrotic state as reflected in the expression profiles. Moreover, inhibition of the highly expressed Jun/Jak/Stat pathway was found to reduce adhesion formation and was proposed as a novel preventative strategy [92].

The role of macrophages in adhesion formation is somewhat controversial with some groups finding a reduction in adhesions in macrophage depleted mice [93], while others reporting an increase in adhesions when macrophages were depleted through chemical means [75]. Furthermore, mesenteric mesothelial cells are thought to transition to a macrophage phenotype post-injury [94] so adding to this complexity. Using a mouse post-operative adhesion model, Hoshino et al. found that macrophages formed aggregates at sites where of adhesion development [95]. This event was regulated by CCR8, a receptor specifically upregulated on peritoneal cavity macrophages but not bone-marrow derived macrophages. Using macrophage *CCR8* deficient mice or an inhibitor of its ligand, CCL1, they demonstrated a significant reduction in the incidence of adhesions [95]. Importantly, CCL1 is produced by mesothelial cells and macrophages and known to upregulate expression of PAI-1 resulting in reduced fibrinolysis. Furthermore, inhibition of PAI-1 is found to reduce F4/80+ macrophage influx and inhibit adhesion formation [75]. Recently, Zindel et al. showed that following severe ischaemic injury, GATA6+ peritoneal cavity macrophages form superaggregates between tissues that act as precursors to adhesions [96].

**Figure 1:**
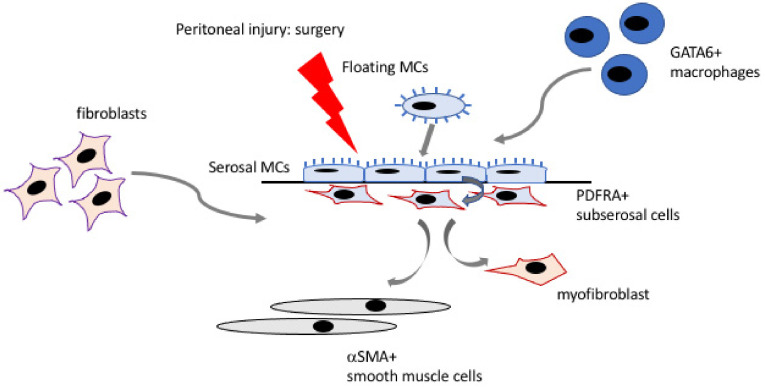
Schematic demonstrating the different cellular contributions to adhesion formation.

Mesothelial cells have been proposed as another important source of fibrogenic cells that mediate adhesion formation through a process of MMT. Using human adhesion samples, Foster and colleagues demonstrated that a small portion of JUN+ fibroblasts also expressed the mesothelial marker MSLN and the EMT pathway was one of the most significantly upregulated molecular profiles in cultured adhesion fibroblasts [92]. Sandoval and colleagues also analysed human adhesions and showed co-localisation of the epithelial marker, cytokeratin and the mesenchymal marker, αSMA, in spindle-like cells in the subserosa strongly implicating that mesothelial cells had transitioned to become myofibroblasts [97], a feature commonly found in biopsy samples from peritoneal dialysis patients [54]. Using the mouse ischaemic -induced adhesion model, they also demonstrated that mature adhesions contained a sub-population of subserosal cells expressing both myofibroblast, such as αSMA, and mesothelial/epithelial markers, such as WT1 and cytokeratin [97]. Recently, Tsai and colleagues demonstrated that inhibition of mesothelin, reduced adhesion formation after ischaemic injury by using clonal analysis and lineage tracing [98]. Furthermore, they showed that the injured surface mesothelium upregulated HIF1α signalling, and that blocking this pathway reduced adhesion formation. Similar markers and signalling molecules were also found in human adhesions [98]. In addition, Strippoli and colleagues discovered a role for mechanical stress in inducing MMT in mesothelial cells which was driven by Caveolin1 and yes-associated protein (YAP1), suggesting an interplay between biochemical and biomechanical signalling in the development of adhesions [99]. Of relevance, fibrin has also been identified as an inducer of MMT [100] and so the combination of many factors post-surgery is likely to create a favourable local environment to trigger adhesion development. The current understanding of cellular contributions to adhesion formation in response to surgical injury is summarised in Figure 1.

## 3. The Developmental Origin of Mesothelium

Strong evidence supports the notion that mesothelial cells are directly incorporated into and play active roles in developing adhesions in the adult. It is therefore important to consider whether a reactivation of the embryonic MMT program occurs with reappearance of certain differentiation markers and developmental signalling pathways. In order to further understand alterations in phenotype and their molecular regulation, in conjunction with molecular expression profiles of mesothelial cells during adhesion formation, changes in key mesothelial cell markers that occur normally during development are discussed.

Mesothelia in the three serosal cavities (pericardial, pleural, and peritoneal) have their origin in mesodermal tissues that are formed during gastrulation in the embryo. The peritoneal coelomic cavity is formed from the lateral plate mesoderm under influence of ectodermal factors [101]. Previous studies in the chick have demonstrated that the mesothelium in the peritoneal cavity arises from resident progenitor cells, in contrast to the pro-epicardium-derived epicardium of the heart [102]. The formation of the mesothelium that covers the intestine was tracked using GFP-expressing plasmid or retrovirus electroporated into splanchnic mesenchymal tissues before the arrival of a mesothelium, and these labelled cells were found to give rise to the epithelial mesothelial lining. Whether this developmental process takes place similarly in the mouse has yet to be demonstrated. Intriguingly, events described by Winters and colleagues [102] suggest that a transition process where splanchnic mesenchymal cells differentiate into mesothelial cells plays a role in the embryonic development of the mesothelium. However, the detailed molecular mechanisms that drive the formation of the mesothelium have not been described yet, neither in the mouse nor in other vertebrate model organisms.

### Molecular Markers

There is only limited evidence of the expression of specific molecular markers during the early stages of mesothelial development. The earliest specific mesothelial marker expressed from around E9.0 onwards is the Wilms’ tumour protein, WT1 [103,104]. The mammalian *Wt1* gene gives rise to at least 36 potential isoforms and these have roles in transcriptional regulation of mesenchymal-epithelial transition and mesenchymal maintenance in kidney development, and epithelial-mesenchymal transition and the regulation of mesenchymal progeny from mesothelial tissues, including the heart, liver, lungs and intestine [105,106]. Lineage tracing studies have demonstrated that WT1-expressing mesothelial cells contribute during embryonic development to lung mesenchyme [107,108,109], cardiomyocytes and coronary vessels in the heart [108,110,111], hepatic stellate and perivascular cells [112,113], pancreatic stellate cells [114,115], and intestinal vascular and visceral smooth muscle [104,116], as well as mesenteric fat [117]. Especially in the developing epicardium, it has been well established that WT1 controls transition towards mesenchymal cell types via transcriptional regulation of Snail and E-cadherin expression [118]. Similarly, WT1 controls EMT in the embryonic epicardium by regulation of Wnt and retinoic acid signalling pathways [119].

WT1 continues to be expressed in the peritoneal and visceral mesothelium into adulthood [104,115]. In the adult mouse peritoneum, WT1 expression has been reported in a defined population of submesothelial cells [64,120]; however, the cell lineage and molecular processes giving rise to submesothelial cells remain obscure. It is important to note that WT1 expression in mesothelial cells is not uniform [65,115], and levels of WT1 expression are reduced in the adult epicardium and lung and liver mesothelium when compared to embryonic stages [121]. Genetic lineage tracing in the adult mouse have shown that in normal tissue homeostasis and when compared to the embryo, WT1-expressing mesothelial cells do not contribute to the mesenchymal cell types in the lung, heart, liver, and intestine [109,110]. However, one caveat of the adult WT1-based mesothelial lineage systems is the observation that they label less than 100% of the mesothelial cells [32,64]. Inactivation of WT1 in the embryo results in lethality due to renal agenesis since WT1 is a dominant regulator of kidney development [122] and also affects the heart, liver, pancreas, and septum transversum [123,124], while adult ablation of WT1 leads to multi-organ failure, affecting the kidneys, haematopoiesis, bone, visceral fat, pancreas, and heart [115,117].

Mesothelin (MSLN) is a glycophosphatidylinositol (GPI)–linked cell-surface glycoprotein which is expressed in developing and adult mesothelium [30,125,126]. It is not known when MSLN starts to be expressed in the developing mesothelium; however, the Eurexpress mouse embryonic expression atlas demonstrates *Msln* RNA expression at E14.5 in the epicardium and pericardium, as well as diaphragm, visceral mesothelium of the intestinal tract, liver, and bladder but also in smooth muscle components [125]. Using a MSLN-based lineage tracing system, mesothelium was shown to contribute to both visceral and vascular smooth muscle and other mesenchymal components of visceral organs, both during embryonic development but also postnatally [31], suggesting that adult MSLN-lineage labelled mesothelium gives rise to a whole range of fibroblasts and smooth muscle cells in the connective and adventitial tissues of organs housed within the serosal cavities. The significance of MSLN expression for mesothelial functions is unknown since MSLN knockout mice grow and reproduce normally and have no detectable mesothelium-related phenotype [127].

Podoplanin (PDPN) is a transmembrane sialoglycoprotein with mucin-like characteristics, which is found in mesothelia of the epicardium, peritoneum, and liver. Besides expression in mesothelial tissues, PDPN is best known for its presence on lymphatic endothelial cells and in the podocytes of the kidneys [128]. PDPN fulfils its perhaps most critical role via binding to the C-type lectin receptor CLEC-2 and subsequent regulation of platelet aggregation and activation [129]. From E10.5 onwards in the mouse, PDPN was detected in the mesothelial linings of the pericardial region, specifically the epicardium and the pericardio–peritoneal canal but also cardiac mesenchyme that differentiates into myocardium; this expression was maintained at later stages in the pericardial and pleural mesothelial lining [130,131,132]. In the E12.5 mouse embryo, PDPN was detected in the liver mesothelium where it was co-expressed with WT1 [112]. In the adult mouse, PDPN expression is maintained in the epicardium and peritoneal and liver mesothelium [32,126]. Interestingly, a mesothelial phenotype has not been reported after functional loss of PDPN in mice [133,134].

The four-transmembrane glycoprotein m6a (GPM6A) plays an important role in neuronal growth cones [135]. GPM6A was identified as a mesothelial marker in E12.5 PDPN+ liver mesothelium [67] and has subsequently been described in adult mouse liver and peritoneal mesothelium [32,67]. GPM6A-based lineage studies have not been reported, while the GPM6A knockout in mice demonstrated no mesothelial phenotype [136].

The *uroplakin 3b* (*Upk3b*) gene encodes a protein with a single transmembrane domain with glycosylated N-terminus at the apical side, which was found to be expressed in mesothelia of the heart, lung, liver, and intestine [30]. Detailed embryonic expression analysis revealed that *Upk3b* transcripts are found in the developing visceral and parietal mesothelium of the peritoneal cavity and in the pro-epicardial organ of the heart and pericardium in the E9.5 mouse embryo [137]. In subsequent embryonic stages, UPK3B expression consolidates in the mesothelial layers of all serosal cavities. The urothelium, which forms from around E14.5 onwards, also expressed UPK3B. Analysis of loss of UPK3B in a knockout mouse line (*Upk3b^CreERT2/+^*) demonstrated no obvious phenotype in the mesothelial tissues nor in the bladder and ureter [137]. The *Upk3b^CreERT2/+^* mouse line and recently generated transgenic mice carrying a Cre cassette under control of the mouse *Upk3b* gene (*Tg(Upk3b-Cre*)) have not been found to be suitable for genetic lineage tracing studies of the mesothelium or urothelium [137,138].

Additional markers identified as specifically expressed in the mesothelium or epicardium and co-expressed with some of the proteins discussed above include Podocalyxin, Cytokeratin 5/6, ALCAM, Desmin, Ezrin, CD200, HBME-1 (rat and human), UPK1, LRRN4, MUC16, TBX18, and intercellular adhesion molecule 1 (ICAM1) [30,65,139,140,141]. However, it is unknown whether mesothelial cells at the different anatomical positions, and throughout all developmental stages and in the healthy adult, uniformly express all these markers. It has also been questioned whether the various mesothelial cell subsets found share a common embryonic origin or actually represent individual mesothelial cell subtypes [98]. Of note, single cell RNA seq analysis of human peritoneum identified *WT1*, *PDPN*, *MSLN*, *UPK3B*, *LRRN4*, *GPM6A*, *ICAM1*, cytokeratins, and calbindin 2 as mesothelial-specific genes [142].

The expression and function of the main mesothelial molecular markers are summarised in Table 1.

## 4. Further Discussion and Areas of Future Research

To better understand the key cellular and molecular mechanisms that contribute to adhesion formation, there needs to be a clearer knowledge of the contribution and source of peritoneal cells in development, homeostasis, and repair. Although many studies have focused on characterising these cells and determining their role post-surgery, including lineage tracing approaches, the wide range of injury stimuli applied in the various experimental model systems is likely to affect the way peritoneal cells respond. Such heterogeneity could explain some of the disparities in the expression of cell markers and hence in findings reported between different studies. Furthermore, certain marker panels may fail to identify all of a particular cell type and their destinations reliably.

Mesothelial cells have also been isolated from adherent or free-floating ascites, lavage, and exudate sources and often also cultured; as such, these cells as well as subserosal cells have been shown to possess multiple properties [143,144,145]. However, it is not clear whether free-floating cells are the same as adherent cells, if contaminated mesothelial cell populations have been isolated due to lack of additional purification steps or whether selection pressures during culture have influenced the cell population analysed and/or its phenotype. Mesothelial cell markers documented during embryonic development may not be consistent with those in the adult, or there may be a reactivation of developmental processes and hence the same markers re-emerge after peritoneal injury in the adult. Most lineage tracing experiments are performed in mice, so it remains to be clarified if these markers are the same as those found in humans. Both animal and human studies have to a certain extent been hampered by the lack of mesothelial cell specific antibody probes capable of positively identifying mesothelial cells while avoiding contamination with other cells. Clearly further research is needed to clarify the repertoire of markers expressed by different populations of peritoneal cells during development, homeostasis, and post-surgery, in both mice and humans.

Peritoneum covering different tissue and organs may have distinctive characteristics and respond in a particular manner to injurious stimuli. It is proposed that different subpopulations of mesothelial cells and subserosal fibroblasts exist that show varying degrees of marker expression [67,92]. It has been found that liver mesothelial cells change phenotype differently compared with cavity wall mesothelium after repeated chemically-induced injury [32]. Peritoneal fibrosis and mass adhesion formation associated with encapsulating peritoneal sclerosis (EPS), a rare but severe consequence of long-term peritoneal dialysis, is predominately localised to the visceral rather than parietal peritoneum and in mice, heterogeneity has been found between the two populations when cells were isolated and analysed in vitro for gene expression and motility [146]. Local microenvironmental factors may influence molecular expression and cell behaviour in different areas of adult peritoneum. For instance, mesothelial cells on the surface of the ovary, also known as ovarian epithelial cells (OECs), are proposed to be different from mesothelium on the rest of peritoneum. Specifically, they have been found to be relatively uncommitted pluripotential cells reflected through a different growth potential, capacity to change phenotype in response to environmental stimuli, and an ability to differentiate along several pathways [147]. Whether they have adopted to this phenotype in order to perform the function of continuous re-epithelialisation following cyclical ovulation remains to be explored. In addition, subpopulations of WT1-positive mesothelial cells and subserosal fibroblasts in the omentum, a vascularised adipose-rich peritoneal fold, have been found to be particularly high producers of retinoic acid. Importantly, retinoic acid is implicated in the homeostasis of cavity GATA6+ macrophages [120,148]. It is interesting to speculate whether dysregulation of this process is part of the reason for a higher incidence of adhesions reported in obese patients [85]. Furthermore, omentectomy in rabbits results in a greater prevalence of intestinal adhesions after surgery in particular in the presence of infection or mesh implant [149]. Hence it is proposed that the omentum acts as a protective mechanical barrier preventing the formation of detrimental adhesions. However, by characterising mesenchymal cells isolated from experimental omental adhesions, Gomez-Gil and colleagues found that their phenotype and behaviour in culture related to type of adhesions they formed, either adipose rich and highly vascularised or fibrous and populated with myofibroblasts [150]. Local environmental factors such as inflammation, infection, ischaemia, and mechanical stress as well as metabolic status likely determine the type of omental adhesions induced to form post-surgery [151]. Interestingly, injured mesothelium is able to signal to uninjured adjacent mesothelium causing a reciprocal change in cell phenotype and behaviour [152]. In addition, calcium-dependent induction of cell membrane protrusions has been found to mediate extensive connections between mesothelial cells that acts as a trigger for adhesion formation [153]. Therefore, it will also be important to explore the influence of such local environmental factors within the peritoneum as a whole.

Adhesion formation and its consequences remains a huge economic and health-related burden following abdominal/pelvic surgery. Moreover, it is envisaged that this problem is on the rise due to a growing ageing population and wider acceptance of certain abdominal/pelvic surgical practices globally. However, new treatments to prevent or limit adhesion formation have not been forthcoming to the clinic. Recent advances have uncovered a greater complexity of the peritoneum than previously recognised and so it may be timely to readdress some of the conventional thinking and bring together disparate specialities with a common interest in peritoneal biology. By doing so, a more comprehensive understanding of adhesion formation will be developed resulting in the generation of better therapeutic strategies.

## Figures and Tables

**Table 1:** Expression and function of mesothelial molecular markers during development.

Gene Name, Molecule Type	Expression in Peritoneum				Role of Protein
	Parietal	Intestine	Liver	Pancreas	Knockout
GMP6A (Glycoprotein m6a)	Adult [32]		Embryonic, adult [67]		Increase in body fat / body weight [136]
PDPN (podoplanin)	Embryonic [131]		E12.5, adult [32,112,126]		Lymphatic and endothelial phenotype [133,134]
WT1 (Wilms’ tumour protein 1)	Embryonic, adult [103,104,116]	Embryonic, adult [103,104,116]	Embryonic, adult mesothelial and submesothelial [112,113,139]	Embryonic, adult [114,115]	Multi-systemic: kidneys, heart, visceral fat, bone, haematopoiesis, pancreas [114,115,117,122]
MSLN (Mesothelin)	Embryonic, adult [31,125]	Embryonic, adult [31,125]	Embryonic, adult [31,125,126]		No phenotype [127]
UPK3B (Uroplakin 3b)	Embryonic, adult [137]	Embryonic, adult [137]	Embryonic, adult [137]		No phenotype [137]
ALCAM (activated leukocyte cell adhesion molecule)			Embryonic, mesothelial and submesothelial [67,112,139]		
